# Supplementary material for: p53 engagement is a hallmark of an unfolded protein response in the nucleus of mammalian cells
Source: bioRxiv. 2024 Nov 8:2024.11.08.622663. Preprint. [Version 1] doi: 10.1101/2024.11.08.622663 (PMC11581032; doi:10.1101/2024.11.08.622663)

Supplementary Figure 3

bioRxiv preprint doi: https://doi.org/10.1101/2024.11.08.622663; this version posted November 8, 2024. The copyright holder for this preprint (which was not certified by peer review) is the author/funder, who has granted bioRxiv a license to display the preprint in perpetuity. It is made available under aCC-BY 4.0 International license.

A

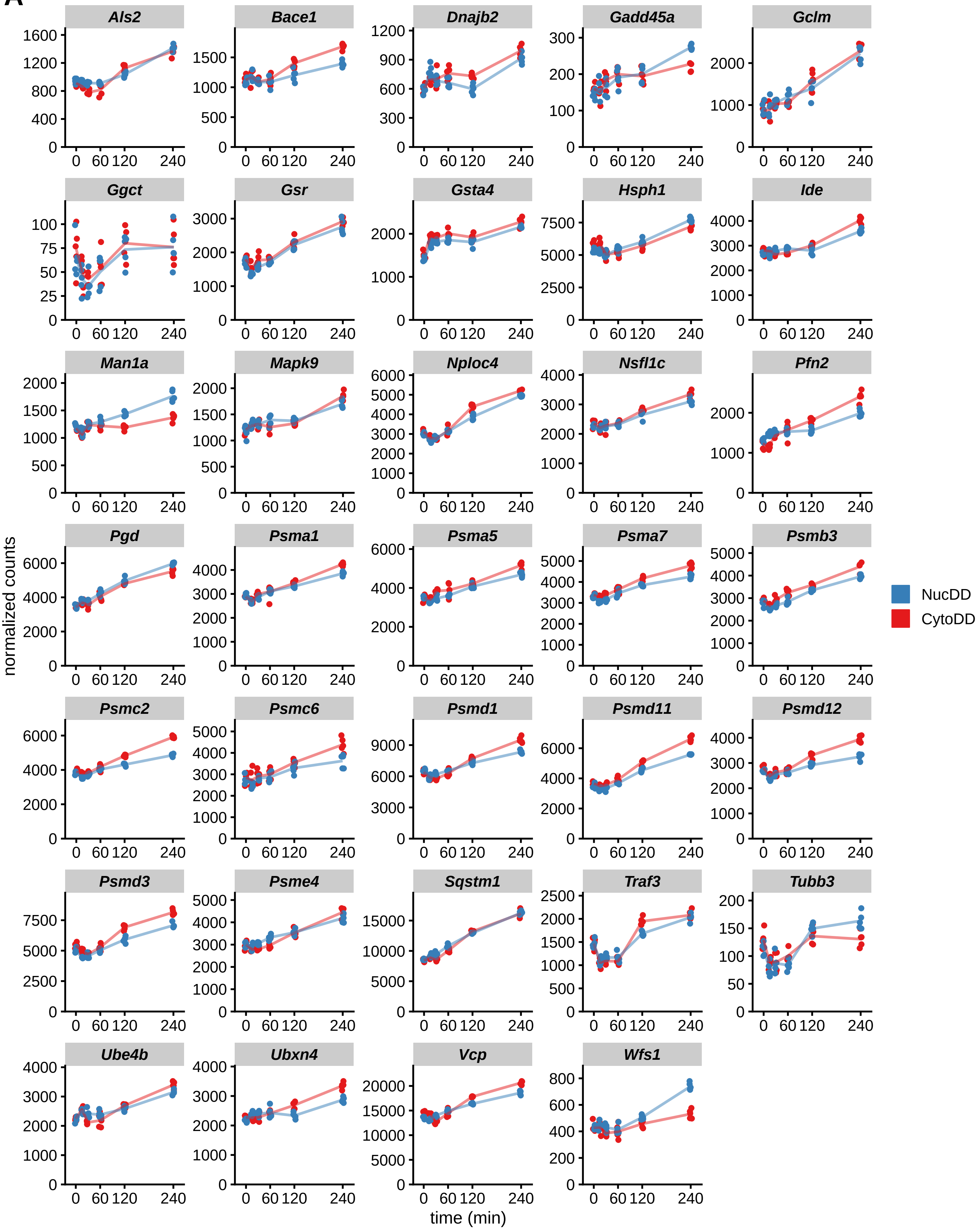

Supplementary Figure 4

bioRxiv preprint doi: https://doi.org/10.1101/2024.11.08.622663; this version posted November 8, 2024. The copyright holder for this preprint (which was not certified by peer review) is the author/funder, who has granted bioRxiv a license to display the preprint in perpetuity. It is made available under aCC-BY 4.0 International license.

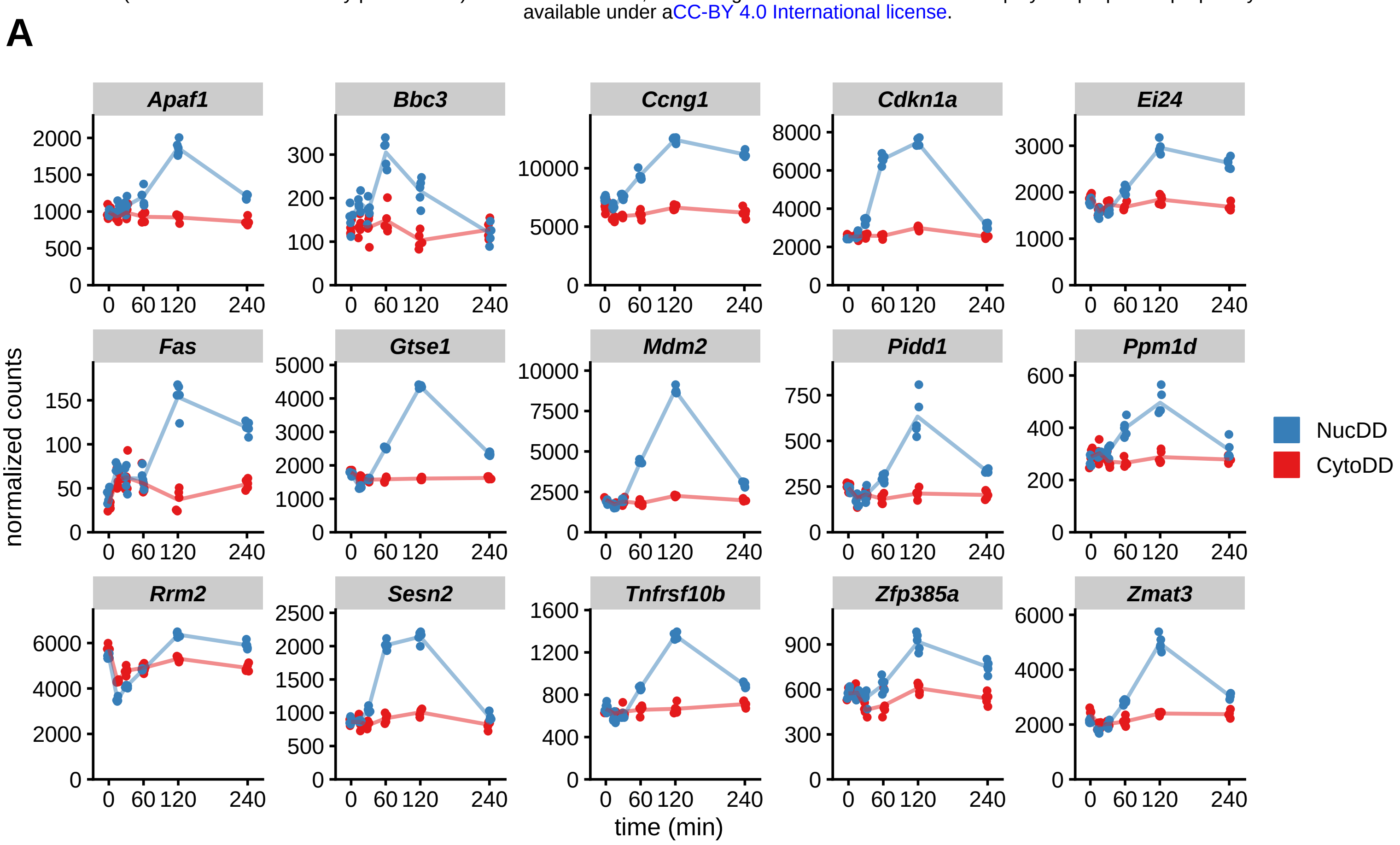

Supplementary Figure 5.

bioRxiv preprint doi: https://doi.org/10.1101/2024.11.08.622663; this version posted November 8, 2024. The copyright holder for this preprint (which was not certified by peer review) is the author/funder, who has granted bioRxiv a license to display the preprint in perpetuity. It is made available under aCC-BY 4.0 International license.

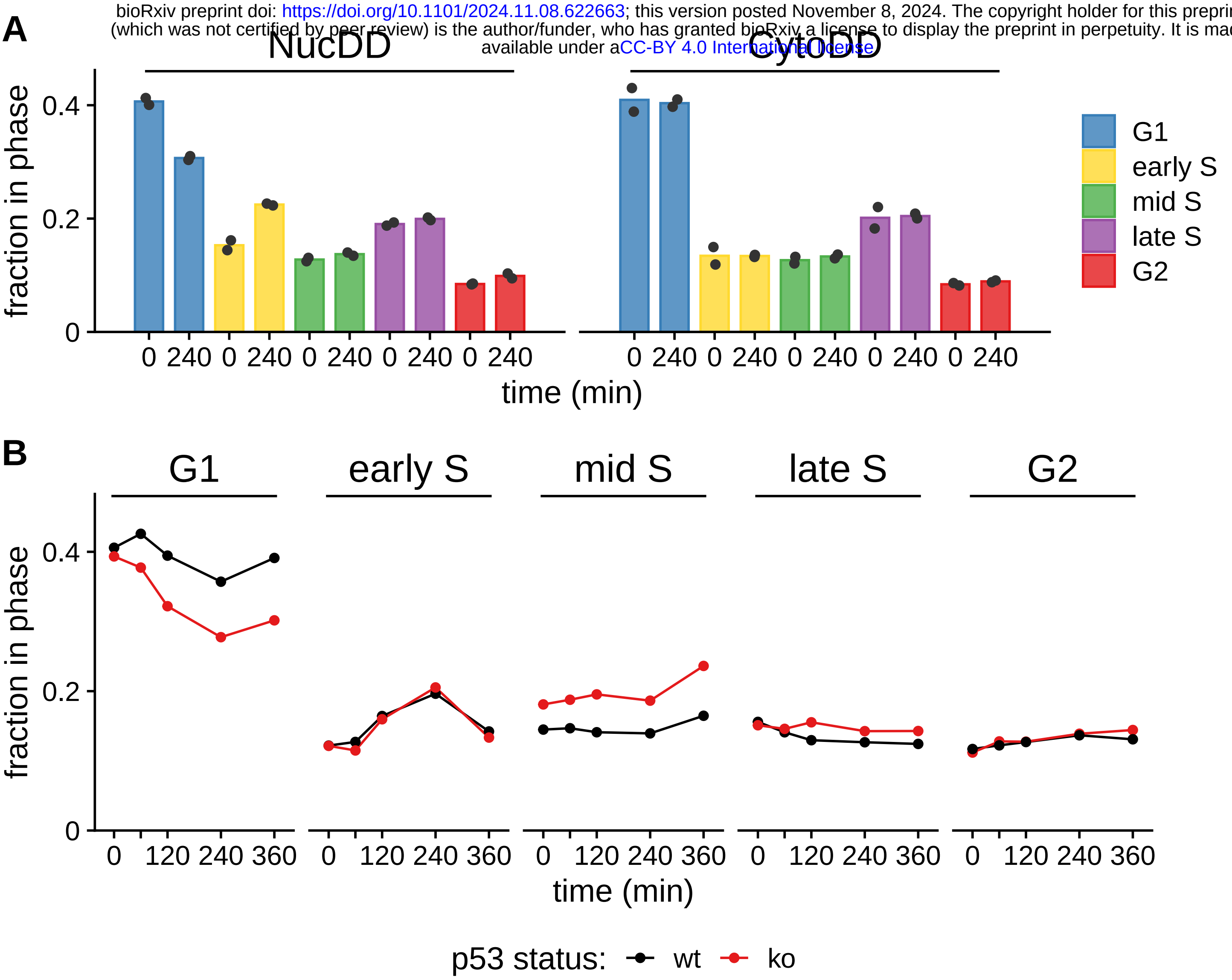

Supplement: Supplement 1 — Supplementary files Supplementary file 1. Log2 fold change and Benjamini-Hochberg (BH) adjusted p-value data for genes identified in NucDD and CytoDD across all time points. Supplementary Figure 3. A. Normalized counts for all genes identified in significantly overrepresented KEGG pathways in cluster C1. Supplementary Figure 4. A. Normalized counts for all ‘p53 pathway’ genes identified from cluster D1 (Fig. 2E) Supplementary Figure 5. A. Mean fraction of NucDD and CytoDD populations gated for each phase in untreated (0 min) or treated (240 min) cells from 2 biological samples. Data from each sample is represented as a point. B. Fraction of p53wt or p53ko NucDD cells in each phase treated with ligand withdrawal for the indicated times. [file NIHPP2024.11.08.622663v1-supplement-1.pdf]
